# New Energy Efficient Multi-Hop Routing Techniques for Wireless Sensor Networks: Static and Dynamic Techniques

**DOI:** 10.3390/s18061863

**Published:** 2018-06-07

**Authors:** Emad Alnawafa, Ion Marghescu

**Affiliations:** Department of Telecommunications, University Politehnica of Bucharest, Bucharest 060042, Romania; marion@comm.pub.ro

**Keywords:** wireless sensor network, clustering, lifetime, stability, throughput, LEACH protocol

## Abstract

The performance of Wireless Sensor Networks (WSNs) faces a number of challenges. Of these challenges, energy consumption is considered a hot research area. Most WSN energy is used in transmitting the data from the sensor nodes either among each other or to a Base Station (BS). For this reason, many routing protocols have been developed to facilitate the data dissemination in the WSNs. One of these protocols, Low Energy Adaptive Clustering Hierarchy (LEACH) has provided a distinctive hierarchical approach that efficiently forwards the nodes data to the BS, but it suffers from increased energy consumption and a significant decline in the network performance in the case of large-scale networks. This paper aims to present a new approach for splitting the whole sensor network into several levels. Thus, every node will be acting accordingly on its position and status. Further, two techniques, a static one and a dynamic one, have been developed to route the data between the levels. The simulation results demonstrated that the proposed techniques prolong the lifespan, improve the stability and raise the throughput of the network compared with the LEACH, the Improved MHT-LEACH (IMHT-LEACH), and the Enhancing DMHT-LEACH (EDMHT-LEACH) protocols.

## 1. Introduction

**Figure 1:**
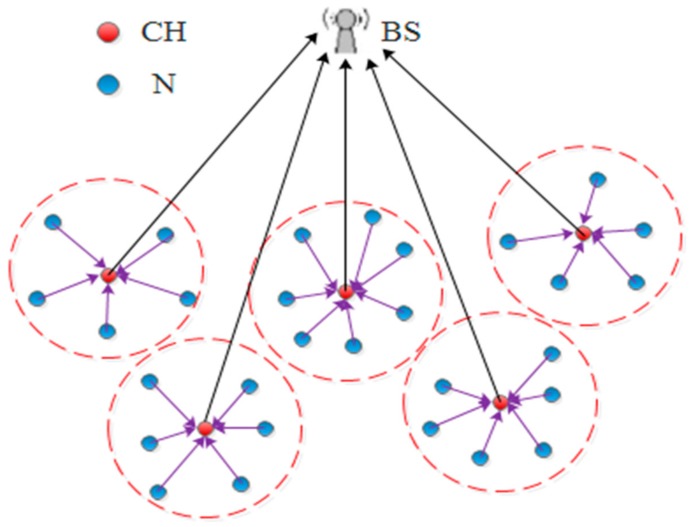
The topology of Wireless Sensor Networks (WSN) using the Low Energy Adaptive Clustering Hierarchy (LEACH) protocol.

Nowadays, Wireless Sensor Networks (WSNs) are attracting the considerable attention of many researchers thanks to their participation in a variety of practical applications [1,2,3]. WSNs have a number of characteristics that make them distinctive from other conventional networks. For instance, the energy sources, the computational capabilities, and the data storages are limited [4,5]. Hence, the characteristics of WSNs must be taken into account during their use. Many routing protocols have been introduced to convey the collected data of WSN to a Base Station (BS), which in turn, forwards it to the end-user [6,7]. In this context, a group of these protocols adopted hierarchical mechanisms for delivering the sensor data to the BS. The Low Energy Adaptive Clustering Hierarchy (LEACH) protocol [8] is one of these protocols which has attained widespread acceptance in this domain. The main concept of the LEACH protocol is to split the whole area of the sensor network into clusters. Thus, every cluster combines an aggregator called a Cluster Head (CH) that is able to collect sensed data from other cluster member nodes. Figure 1 presents the topology of the sensor network within the LEACH protocol. In the LEACH protocol, the network’s lifespan is also divided into specific durations called rounds and each round includes two phases. The first phase is the set-up process, which is dedicated to picking out the network’s CHs and forming the clusters. For this reason, the whole sensor nodes choose in advance a random number between 0 and 1. Then, every node compares its random number with a threshold value *T*(*n*). Consequently, if its number is less than the *T*(*n*), this node begins working as a CH for the round in progress. It shall be noted that the *T*(*n*) value can be calculated by the Equation (1) [8]:

(1) $$T\left(n\right)=\{\begin{array}{c} \frac{p}{1-p\left(r\;mod\frac{1}{p}\right)}\;if\;n\in G\\ 0\;otherwise \end{array}$$

In Equation (1), the *p* parameter denotes the percentage of CHs that has to exist in the network, the *r* parameter indicates the round’s number, and the *G* parameter refers to the set of sensor nodes, which have not been CHs in the last 1/*p* rounds.

Subsequently, all CHs start establishing their clusters by broadcast announcements. Besides, every normal node chooses its CH based on the Received Signal Strength Indicator (RSSI) and then sends a Join-Request message (JOIN-REQ) to it. Finally, the CH of each cluster creates a Time- Division Multiple Access (TDMA) schedule and transmits it to all members of the cluster. Thus, every cluster node is able to send the data in its particular time according to the TDMA schedule. After the end of the set-up phase, the second phase that is called the steady-state phase will be started. In the steady-state phase, the cluster’s member nodes begin their working. Consequently, the normal member nodes (Ns) start gathering the data from its surrounding and forward it to its CH. The collected data, which is received by the CHs, will be compressed and transmitted directly to the BS. To obviate the interference problem between the clusters, the clusters can use different solutions such as different Code-Division Multiple Access (CDMA) codes.

A number of pros are achieved through using the LEACH protocol in WSN. For instance, the election process gives equal opportunities for all the nodes of the network to become CHs. Furthermore, the TDMA schedule has been proposed in this protocol in order to manage data transmitting within the cluster. Additionally, it allows using active/sleep mode for the Ns within the cluster. Conversely, a number of cons have appeared for the LEACH protocol. For example, routing the sensed data through a large-scale network using the LEACH protocol is an inappropriate way, bearing in mind that it uses a single-hop method to transmit the CH data to the BS. Consequently, the amount of the energy consumption, especially in case of the nodes that are far from the BS, will rise significantly. At the same time, the CH election process, which is used in the LEACH protocol does not consider the energy level in the nodes. Such problems will negatively affect the lifetime and the performance of the sensor network [9].

The Improved MHT-LEACH (IMHT-LEACH) protocol was proposed in [10] to provide an approach for transmitting the CH data from sensors over an unlimited number of levels toward the BS. The length of each level is *d_o_*/2. The IMHT-LEACH protocol is also considered as an improvement approach for the Multi-Hop LEACH (MHT-LEACH) protocol [11]. It supposes that the network lifetime is split into rounds and each round comprises of three primary phases: the initial phase, the announcement phase and the routing phase. In the initial phase, the clusters are formed by using the same approach that is used in the LEACH protocol. Thus, the CHs of the network will be elected and other sensor nodes will join to their clusters. In the announcement phase, all the CHs of the network declare themselves to each other. The previous phase gives sufficient information to the CHs in order to create their routing tables (RTs). In the routing phase, each CH decides to route its data directly or through another CH toward the BS based on the available routes distances in its RT. Obviously, the routes that have lowest distances will be chosen. The IMHT-LEACH protocol achieved advantages related to reducing the energy consumption and arranging the data transmitting process from the CHs to the BS. On the other side, it did not show interest in: the clusters formation, the transmitting the data within the clusters and the energy load that will appear due to using the same routes along the round.

The Enhancing DMHT-LEACH (EDMHT-LEACH) protocol was proposed in [12]. The main purpose of the EDMHT-LEACH protocol is to upgrade the performance of the Dynamic Multi-Hop LEACH (DMHT-LEACH) protocol [13]. Similar to the IMHT-LEACH protocol, the sensor nodes are distributed over the levels around the BS and the length of each level is *d_o_*/2. Further, it splits the network lifetime into rounds. The main idea of the EDMHT-LEACH approach is built on dividing each round into four phases: the initial phase, the announcement phase, the selection phase and the routing phase. In the initial phase, an approach was proposed to control the number of sensor nodes within the clusters in order to relieve energy loads on the CHs. Moreover, a new designation (Independent Nodes—INs) and tasks have been given to the sensor nodes that can’t join any network cluster. In the announcement phase, all the CHs and the INs proclaim themselves to each other to facilitate creating the RTs. In the selection phase, the RTs of the CHs and the INs will be established. Finally, a cost function is evaluated and used in the routing phase to arrange the routes of the RTs based on their costs. Here, the lowest cost routes will be initially chosen to be used. During the round, the RTs of the CHs and the INs will be updated and the initial routes may be changed during the same round. The EDMHT-LEACH approach has reduced the total energy consumption of the network and distributed the energy load among different next hops. Hence, the lifespan of the sensor network is prolonged and its stability is increased. On other hand, the EDMHT-LEACH protocol did not pay attention to the data transmitting of the sensor nodes within the clusters. Thus, the dynamic routing approach is only used outside the clusters, whereas the direct data transmitting is used within the clusters as in LEACH protocol.

In this paper, we aim to prolong the sensor network lifetime and its performance. Therefore, a new general approach is proposed for distributing all the deployed sensor nodes into levels around the BS. Moreover, we suppose that each cluster will be divided into two levels around the CH too. The main reason behind the previous assumption is to minimize the data transmission distances as much as possible between the nodes themselves and from them to the BS. The network lifetime is split into rounds, and it is assumed that the sensor nodes will belong to one of three groups (CHs, INs and Ns) and have different responsibilities from one round to another. In this context, we expect that the energy load will be distributed among all the network member nodes, which will increase the stability of the network. Further, new full routing mechanisms will be utilized to convey the data intra-cluster and inter-cluster. One of these mechanisms belongs to the static class of routing algorithms, while the second mechanism belongs to the dynamic one. In the case of the new approaches, the routing data process becomes more organized and dependent on the coordinates and energy status of every sensor node. The static routing mechanism proposes a static approach based on choosing the routes that have the shortest distances to be used along the duration of the round. By the static mechanism, we look forward to selecting the lowest distance routes to minimize the energy dissipation needed to transmit the sensor data. For considering the energy loads on the next hop nodes, a dynamic mechanism is proposed. This approach considers switching between the available routes depending on a cost equation that was defined for this purpose. By proposing the dynamic approach, we seek to reduce the energy consumption and distribute the energy load on different routes during every round.

This paper is organized as follows: Section 2 describes some protocols presented in the related research. Section 3 is dedicated to analyzing the whole approaches of the proposed ideas. Section 4 presents the evaluation of the proposed algorithms performance by means of simulation and discusses the obtained results. Finally, Section 5 concludes the paper.

## 2. Related Works

Due to its outstanding success, LEACH protocol has gained much popularity in the WSN research field. Accordingly, one can find many studies which have proposed new techniques for the data routing in WSN, by modifying the LEACH protocol in order to mitigate its drawbacks. This section presents some of these approaches as related work to the approaches developed by us. In this context, it would be useful to mention that all of these approaches have succeeded to upgrade the WSN performance in comparison with conventional LEACH protocol. A part of these protocols has modified the set-up phase of the LEACH protocol, while the remaining part was interested in enhancing the steady-state phase. 

The LEACH-Centralized (LEACH-C) protocol was proposed in [14]. It is described as a centralized protocol due to all decisions being issued by the BS and then forwarded to the sensor nodes. For instance, electing the CHs of the network and managing the clusters are accomplished by the BS. The expected result of the LEACH-C protocol is to have an excellent distribution of nodes among the clusters. The LEACH-Deterministic Cluster Head Selection (LEACH-DCHS) protocol, which is presented in [15], aims to prolong the network lifetime. Accordingly, two modifications have been proposed. The first one is adjusting the threshold equation, which is used in electing the CHs of the network by taking into account the residual energy in the sensor nodes. The second modification is introducing a new definition for the network lifetime. The main idea of the Threshold-LEACH (T-LEACH) protocol was analyzed in [16]. In this protocol, the CHs of the network are chosen based on a threshold energy scheme. Moreover, the CHs are used for a fixed number of rounds. This situation is continued until the remaining energy of the CH becomes less than the threshold energy. If this happens, a new CH will be elected. 

The Unequal Clustering LEACH (U-LEACH) protocol was proposed in [17] and aims to reduce the energy consumption in a single hop communication, such as the LEACH protocol. In single-hop fashion, the CHs data is transmitted directly into the BS. The previous approach increases the energy consumption in the CHs, which are located far from the BS. For this reason, the U-LEACH supposes that the clusters size will be unequal and that it will be smaller as we move away from the BS. To solve the unbalanced clusters in the conventional LEACH protocol, the LEACH-Balanced (LEACH-B) protocol was proposed in [18]. This protocol takes into consideration the desired percentage of the nodes within the clusters, as well as it takes into account the residual energy of the nodes for electing the CHs and forming balanced clusters. For minimizing the transmissions distances over the network, the MHT-LEACH protocol was proposed in [11]. It splits all CHs into two levels. The first one is the internal level that includes all the CHs whose distances to the BS are less than a specific threshold. The second one is the external level which includes the CHs whose distances to the BS are equal or larger than the threshold. The data from any CH of the external level are routed to the BS by means of an appropriate CH from the internal level. 

A solution, Solar Aware-LEACH (s-LEACH), for adding an external energy source for the sensor nodes was discussed in [19]. The solution is based on using the solar power. All sensor nodes transmit their solar power status and residual energy to the BS, which, in turn, chooses the nodes that have a good power status and higher residual energy to become CHs. A new protocol for selecting the CHs of the network, the Advanced-LEACH (ALEACH) protocol, was suggested in [20]. According to this protocol, the threshold value is based on two terms: a general state probability and a current state probability. Thus, their combination provides the threshold value for each round. The DMHT-LEACH protocol developed and analyzed in [13] for enhancing the CHs data routing in the IMHT-LEACH protocol. It suggests a dynamic approach for conveying the CHs’ data over the levels toward the BS. For choosing their routes during the rounds, the CH uses a dynamic approach, which considers the residual energy of the CHs and the distances between them. 

## 3. Proposed Techniques

This paper introduces two techniques for transmitting the sensor data to the BS: a static and a dynamic technique. Thus, we suppose that the lifetime of the network is split into rounds. Every round comprises of four phases: the initial phase, the announcement phase, the tables preparation phase, and the routing phase. It is worth mentioning that the basic difference between the two techniques appears in the routing phase. This study assumes that the nodes of the network are all stationary and scattered randomly along the environment. Additionally, they will be initiated by a limited quantity of energy. To compute the energy receding in the network nodes, we use the energy dissipation model, which was proposed in [14]. By doing so, the amount of energy which is required to transmit and receive *k*-bit packets can be calculated using the following equations, respectively [14]:

(2) $$E_{Tx}\left(k,\;d\right)=\{\begin{array}{c} E_{elec}\times k+\varepsilon_{fs}\times k\times d^{2}\;,\;d<d_{o}\\ E_{elec}\times k+\varepsilon_{mp}\times k\times d^{4},\;d\geq d_{o} \end{array}$$

(3) $$E_{Rx}\left(k\right)=E_{elec}\times k$$

Here, the *E_elec_* is the energy that is consumed per bit in order to process the data that will be transmitted and received along the network. The $\varepsilon_{fs}$ indicates the free space propagation model, which is utilized in evaluating the energy needed if the transmission distance is less than a threshold distance (*d_o_*). The $\varepsilon_{mp}$ refers to the two-ray propagation model and is used when the transmission distance is equal to or longer than *d_o_*. The value of the *d_o_* can be found as follows [14]:

(4) $$d_{o}=\sqrt{\varepsilon_{fs}/\varepsilon_{mp}}$$

### 3.1. Initial Phase

In this phase, the network’s CHs are all elected. For this reason, every sensor node selects a random number between 0 and 1. Then, this random value is compared with a variable threshold *T*′(*n*). If it is less, that node will be a CH for the period of the current round. The value of *T*′(*i*) can be calculated using Equation (5), which is proposed in [21]. We prefer to use this equation bearing in mind that it is considered a modification of Equation (1). It takes into account the residual energy in the sensor nodes during the selection of the CHs, which in turn reduces the random character of this process [21]:

(5) $$T^{\prime }\left(i\right)=\{\begin{array}{l} max\left(\frac{p}{1-p\left(r\;mod\frac{1}{p}\right)}\times \frac{E_{residual}}{E_{max}},T_{min}\right)\;\forall_{i}\in G\\ 0\;\forall_{i}\notin G \end{array}$$

Here, the *E_residual_* is the amount of energy, which the node still has after a period of working. *E_max_* denotes the amount of energy incorporated in a node before starting its activity. *T_min_* refers to the minimum value of the threshold, which can be used if the *E_residual_* value has descended to low values. After finishing electing the CHs, the clustering formation process begins. Hence, in what follows, we introduce a number of concepts useful for clusters formation and the network topology.

#### 3.1.1. Number of Member Nodes of the Cluster (*N_o_*)

The proposed protocol supposes that each cluster will have a limited number of member nodes, which means that each CH can relate to a specific number of member nodes and cannot surpass it. The main target for this assumption is to make a balance between the clusters. Thus, the expected number of the CHs for a network owns *J* sensor nodes will be [12]:

(6) $${\mathrm{Number}\;\mathrm{of}\;\mathrm{the}\;\mathrm{CH}}_{s}=\;\overset{J}{\underset{n=1}{\sum }}p\times 1=J\times p$$

Based on the above, the limited number of sensor nodes that can be joined to each CH will be [12]:

(7) $$N_{O}=\frac{J}{{\mathrm{Number}\;\mathrm{of}\;\mathrm{the}\;\mathrm{CH}}_{S}}$$

For creating the network clusters, all the CHs announce themselves to other nodes of the network via broadcast messages. Each announcement message includes the CH identification (CH ID) and the coordinates of its position. On the other side, the normal sensor node, who receives this message, calculates the distance to the CH. Afterward, it inserts this value in the selection table (ST), which is later used to choose the closest CH. Moreover, the proposed approach assumes that the sensor node is only able to join the CHs, whose distances are less than *d_o_*. Hence, the distances that are equal or greater than *d_o_* will be neglected from the ST. Usually, the sensor node transmits a JOIN-REQ message toward the CH that owns the lowest distance value in the ST. The CH, in turn, checks the availability in the TDMA schedule. Once the CH finds a free slot, it allocates and sends back a response to the node. Else, the CH transmits a refusal message. In the previous case, the node goes to the second lowest value in the ST and transmits another JOIN-REQ message to the corresponding CH and so on [12].

#### 3.1.2. Independent Nodes (INs)

The result of the previous assumption is that a large part of the nodes has joined the existing clusters of the network. The remaining part of sensor nodes have not been able to join any cluster and they will be called INs. The proposed protocol grants the INs the ability to route their data to the BS through other INs or CHs. Moreover, any IN can aid other INs or CHs to route their data to the BS. Conversely, the INs are unable to contact the regular nodes like the CHs [12]. Finally, the detail procedure of the clustering process, which takes place at the beginning of every round, is given by the pseudo-code in Algorithm 1.
**Algorithm 1:** Clustering process
*X* ∈ group of the deployed sensor nodes 
*R*   a random number between 0 and 1
*T*′(*i*)  threshold value
*N*′   number of the nodes that joined to the CH
*N_o_*   maximum number of the member nodes that can join to the CH
ST   selection table1:begin2:for each (*X*)3:  *X* selects *R*4:  if (*R < T*′(*i*)) then5:     *X* becomes CH in this round; 6:  else7:     *X* remains a regular node waiting to join a cluster;8:  end if9:end for10:for each (CH) 11:  Broadcast a message advertising its status to *X*s;12:  Wait for a *Join-Request messages* from *X*;13:   if (*N*′ > *N_o_*) then14:    Send a refusal for *Join-Request message* from *X*;15:   else16:    Send an acceptance for *Join-Request message* from *X*;17:    Allocate a time slot in the TDMA schedule and send it to *X*;18:   end if19:end for20:for each (*X*)21:  Wait for advertising messages from CHs;22:  if (*available_routes_distances_in _ST* >= *d_o_*) then23:    *X* becomes IN in this round;24:  else25:    Send a *Join-Request message* to the CH;26:    wait a response for a *Join-Request message*;27:  end if 28:    if (*X*_joined_to _CH) then29:      *X* becomes *N* in this round; 30:      Wait for TDMA shedule from CH;31:    else32:      Repeat Steps 22:27;33:    end if34:end for

#### 3.1.3. Network Leveling

**Figure 2:**
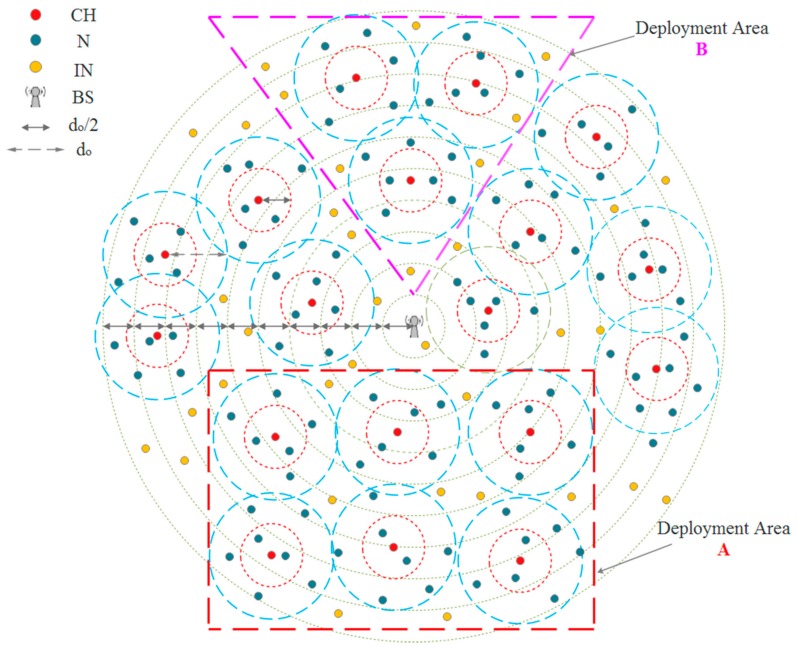
The general topology of a WSN using the proposed approach.

According to the proposed approach, the whole architecture of a sensor network is split into a number of levels around the BS, the length of each level being *d_o_*/2. All the CHs and the INs will be distributed into levels based on their distances to the BS (Figure 2). For example, the CH located at a distance less than *d_o_*/2 from the BS will belong to the first level, whereas the CH with a distance equal or larger than *d_o_*/2 but less than *d_o_* will belong to the second level and so on. Furthermore, it supposes that each cluster will be divided also into two levels around the CH, using the same procedure (Figure 2). By doing so, all the CHs, INs, and Ns will have their specific levels, and most of the data transmissions, which occur between the sensor nodes in any two successive levels, will stay within transmission distances less than *d_o_*. We expect this solution will positively reflect on the amount of consumed energy, which can be calculated using Equation (2). 

Additionally, we suppose that each cluster owns a cluster ID, which distinguishes it from other clusters. Usually, the Ns use the cluster ID to forward the sensed data to their CH. Figure 2 shows a general topology of a WSN using the proposed approach. Indeed, the proposed technique can be applied into deployment areas of sensor nodes of different shapes (parts of the general topology) and regardless of the BS position either inside or outside of the deployment area. For example, the deployment areas A and B represent two different situations that could be encountered in practice. So, even if we suppose that the sensor nodes are only deployed on the rectangular area (A) or on the triangular area (B), the same general leveling approach to distribute the nodes into levels based on their distances to the BS is used.

### 3.2. Announcement Phase

**Figure 3:**
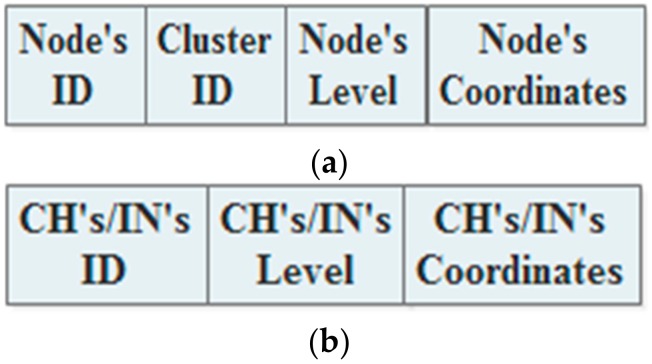
The announcement messages in the static approach: (**a**) the Normal member node (N)’s message; (**b**) the Cluster Head (CH)’s/ Independent Node (IN)’S messages.

During the network lifetime, all the CHs, INs, and Ns are regularly declaring themselves to each other by broadcasting announcement messages. In the static approach, it is assumed that the different kinds of nodes use different types of broadcasting messages. Usually, they only use the messages at the beginning of every round. Hence, the Ns broadcast messages that include their IDs, cluster IDs, levels, and coordinates, is shown in Figure 3a. Furthermore, the CHs and INs broadcast announcement messages containing their IDs, levels, and coordinates, is shown in Figure 3b.

**Figure 4:**
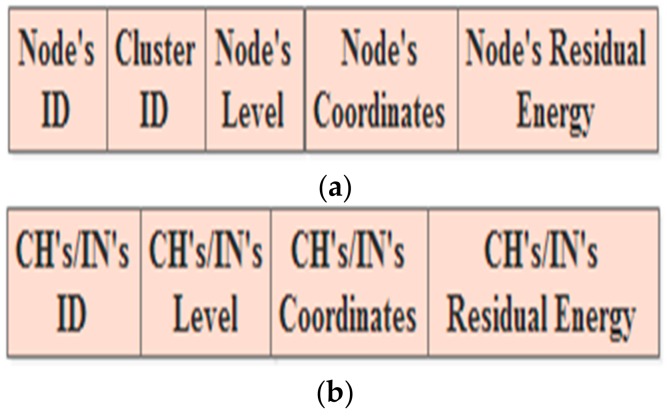
The announcement messages in the dynamic approach: (**a**) the N’s message; (**b**) the CH’s/IN’S message.

**Figure 5:**
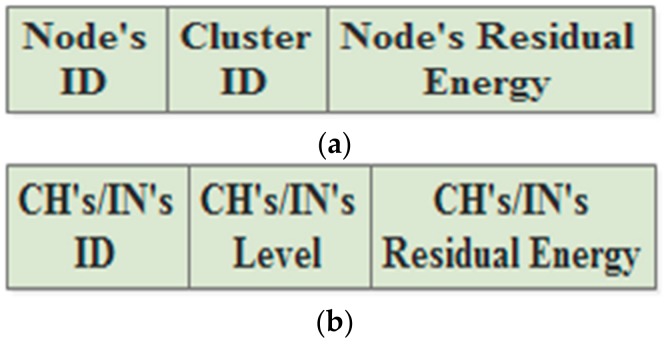
The updated messages in the dynamic approach: (**a**) the N’s message; (**b**) the CH’s/IN’S message.

The dynamic approach specifies two types of broadcasting messages. The first one is used at the beginning of the round. Thus, the Ns of all clusters broadcast message comprising their IDs, cluster IDs, levels, coordinates, and residual energy, is shown in Figure 4a. In turn, the CHs and INs broadcast messages containing their IDs, levels, coordinates, and residual energy, is shown in Figure 4b. During the round period, other broadcasting messages are used in order to update the RTs. The updated messages of Ns usually include their IDs, cluster IDs, and residual energy (Figure 5a) and the updated messages of the CHs and INs contain their IDs, level numbers, and residual energy (Figure 5b).

### 3.3. Tables Preparation Phase

As it results from the previous phase, all the network nodes are able to generate their RTs. In both approaches, the N, which is located on the second level in each cluster, creates its RT. It usually uses the announcement messages, which are received from the Ns in the first level. Afterward, it transmits a JOIN-REQ to the source of the message. Subsequently, the N that receives the request checks the availability in its TDMA scheme. If there is an empty slot, it allocates for the requester. Then, it transmits its response to it. Thus, the Ns that send approvals to the requests can be added to the RTs.

**Figure 6:**
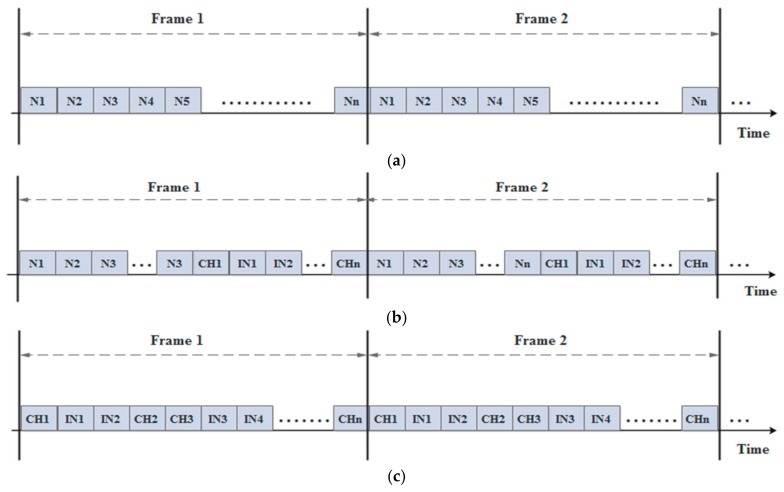
The Time- Division Multiple Access (TDMA) schedule of (**a**) N; (**b**) CH; and (**c**) IN.

The CHs and INs, which are located on the second and upper levels, generate their RTs based on the announcement messages that have been transmitted from lower levels. Accordingly, all messages, which are received from the same and upper levels, will be neglected. Thus, the CHs and INs transmit the JOIN-REQ messages to each other and they wait for the approval to enable them to create their RTs. Figure 6 shows the TDMA schedules for the different types of the network’s nodes during one round. Obviously, the TDMA schedule of the N is only dedicated for its neighboring nodes at the same cluster, whereas the TDMA schedule of the CH has been divided into two sections: the first part is allocated to its member nodes and the second part is allocated to other neighboring CHs and INs. Finally, the slots of the TDMA schedule of the IN are only assigned to the neighboring CHs and INs.

### 3.4. Routing Phase

After completing the previous three phases, the network becomes ready to route the sensed data. Each technique uses a particular approach for routing the network data, as is described in the next paragraphs.

#### 3.4.1. Static Multi-Hop Routing Technique (SMR)

This section provides a static technique for transferring the data along the network levels toward the BS. As its name implies, this technique selects static routes from the network nodes to the BS at the beginning of each round and continues using it until the round is finished. By doing so, the proposed technique assumes that the data transmissions are divided into two groups: the intra-cluster routing and inter-clusters routing. Below, the two groups are debated in detail. Eventually, the node’s energy recedes based on the transmission distance, which is calculated by Equation (2).

- Intra-cluster Routing

As mentioned above, all the network clusters are split into two levels around the CHs, where the length of each one is *d_o_*/2. Each N computes its distance to the CH (*N_D-CH_*) to determine its level. The Ns of the first level send their collected data to the CH directly. On the other hand, the Ns of the second level use their RTs to select their routes. Here, the minimum distance value in the RT will be selected, which may be the distance value to the CH or to another neighboring node in the first level. The details of the intra-cluster routing process are giving by the pseudo-code in Algorithm 2.
**Algorithm 2:** The intra-cluster routing for N
NHN  next hop node (N) to the CH
RT    routing table
*N_D-CH_*  indicates the N distance to the CH
*N*_*D-N*1_  indicates the minimum distance in the RT for NHN within the level 11:begin2:if (*N_D-CH_* >= *d_o_*/2) then3: Create its RT;4:  if (*N_D-CH_ <= N*_*D-N*1_) then5:    Transmit the data directly to the CH using *N_D-CH_*;6:  else 7:    Transmit the data to the NHN in the level 1 having the minimum distance (*N*_*D-N*1_);8:  end if9:else10:   Transmit the data directly to the CH using *N_D-CH_*;11:end if

- Inter-clusters Routing

This group includes all the CHs and INs of the network, which are distributed along the levels around the BS. Thus, the CHs and INs of the first level do not need to create RTs. Additionally, they can forward their data to the BS directly. On the other hand, the CHs and INs that are on the second level or more have to establish their RTs. Based on their RTs, the CHs and INs can determine the next hops to the BS. Clearly, the CH’s and the IN’s data will be transmitted to the minimum distance destination, which could be to the BS or one member of RT. The details of the inter-cluster routing processes for the CH and the IN are giving by the pseudo-code in Algorithm 3 and Algorithm 4 respectively.
**Algorithm 3:** The inter-cluster routing for CH
NHI    next hop node to the BS, which might be a CH or IN
*CH_D-BS_*  indicates the CH distance to the BS
*CH_D-L_*_1_  indicates the minimum distance in the RT for NHI within the level 1
*CH_D-Ln_*  indicates the minimum distance in the RT for NHI within the level n1: begin2:if (*CH_D-BS_* >= *d_o_*/2) then3:   Create its RT;4:    if (*CH_D-BS_ >= d_o_*) 5:     if (*CH_D-BS_ > CH_D-Ln_*)6:      if (*CH_D-Ln_ >= d_o_*) then7:       Transmit the data to the NHI in the level n having the minimum distance (*CH_D-Ln_*); the second part of the equation 2 will be used;8:      else 9:       Transmit the data to the NHI in the level n having the minimum distance (*CH_D-Ln_*); the first part of the equation 2 will be used;10:      end if11:     else12:       Transmit the data directly to the BS using *CH_D-BS_*;13:     end if14:    else15:     if (*CH_D-BS_ > CH_D-L_*_1_) then16:      Transmit the data to the NHI in the level 1 having the minimum distance *(CH_D-L_*_1_);17:     else18:      Transmit the data directly to the BS using *CH_D-BS_*;19:     end if20:    end if21:else22:   Transmit the data directly to the BS using *CH_D-BS_*;23:end if 
**Algorithm 4:** The inter-cluster routing for IN
NHI   next hop node to the BS, which might be a CH or IN
*IN_D-BS_*  indicates the IN distance to the BS
*IN_D-L_*_1_  indicates the minimum distance in the RT for NHI within the level 1
*IN_D-Ln_*  indicates the minimum distance in the RT for NHI within the level n1: begin2:if (*IN_D-BS_ >= d_o_*/2) then3:  Create its RT;4:   if (*IN_D-BS_ >= d_o_*) 5:     if (*IN_D-BS_ > IN_D-Ln_*)6:      if (*IN_D-Ln_ >= d_o_*) then7:       Transmit the data to the NHI in the level n having the minimum distance (*IN_D-Ln_*); the second part of the equation (2) will be used;8:      else 9:       Transmit the data to the NHI in the level n having the minimum distance (*IN_D-Ln_*); the first part of the equation (2) will be used;10:      end if11:     else12:      Transmit the data directly to the BS using *IN_D-BS_*;13:     end if14:   else15:     if (*IN_D-BS_ > IN_D-L_*_1_) then16:       Transmit the data to the NHI in the level 1 having the minimum distance *(IN_D-L_*_1_);17:     else18:       Transmit the data directly to the BS using *IN_D-BS_*;19:     end if20:   end if21:else22:   Transmit the data directly to the BS using *IN_D-BS_*;23:end if 

In summary, the SMR technique provides an approach that is focused on minimizing the data transmission distances, either inside or outside the clusters, through distributing all the different types of sensor nodes into levels. The SMR also controls the number of sensor nodes within each cluster for minimizing the energy consumption in the CHs. In addition, it allows the CHs and the INs to transmit their data to the BS through other CHs or INs, which are in the lower levels. Consequently, the opportunities for transmitting the data over short distances between the levels have increased. Moreover, the SMR technique makes the data routing process more organized by introducing the idea of creating RT for each node. Accordingly, the node has sufficient information about the next hops and it can forward its data using the shortest route.

#### 3.4.2. Dynamic Multi-Hop Routing Technique (DMR)

As recently debated, the static technique introduces a new static approach for conveying the data of nodes to the CHs, and then to the BS, which completely depends on choosing the routes that have the lowest distances. In this section, a dynamic technique is proposed to be applied to the topology of this network. As its name implies, this technique supposes that all nodes of the network, which own a number of routes in their RTs, can use different routes during the same round. For this reason, all nodes of the network arrange the available routes based on a cost function, which has been developed for this purpose. Like the static technique, this technique is also applied on the intra-cluster and inter-cluster transmissions.

- Intra-clusters Routing

At the beginning of each round, the Ns of the second level in each cluster arrange their RTs based on a cost function that is comprised of two parts: the first part is related to the relative location of the next hop node (NHN) to the CH, while the second part is allocated for the ratio of the residual energy in the NHN. The cost value can be computed using the following equation [12]:

(8) $$C_{NHN}\left(i\right)=C_{NHN-CH}\left(i\right)+\alpha \;\cdot \;C_{NHNE}\left(i\right)$$

The *C_NHN-CH_* parameter refers to the distance cost for the NHN. The *C_NHNE_* parameter indicates the energy cost for the NHN. The variable *i* indicates the member’s number within the set of nodes, which the source node (SN) selected and added to its RT in the current round. Finally, the *α* parameter represents the relative weight of the energy cost part in the cost equation. The first part can be obtained using Equation (9) [12]:

(9) $$C_{NHN-CH}\left(i\right)=\frac{{D_{i}}^{2}}{SN_{D-CH}-NHN_{iD-CH}}$$

The *D_i_* parameter indicates the distance from the SN to NHN. The *SN_D-CH_* parameter refers to the distance between the SN and its CH, while *NHN_iD-CH_* indicates the distance between the NHN and its CH. The second part of the cost function is the energy ratio in the NHN and it can be computed using Equation (10):

(10) $$Energy\;ratio\;=\frac{\;RE_{NHN}}{\;IE_{NHN}}$$

**Figure 7:**
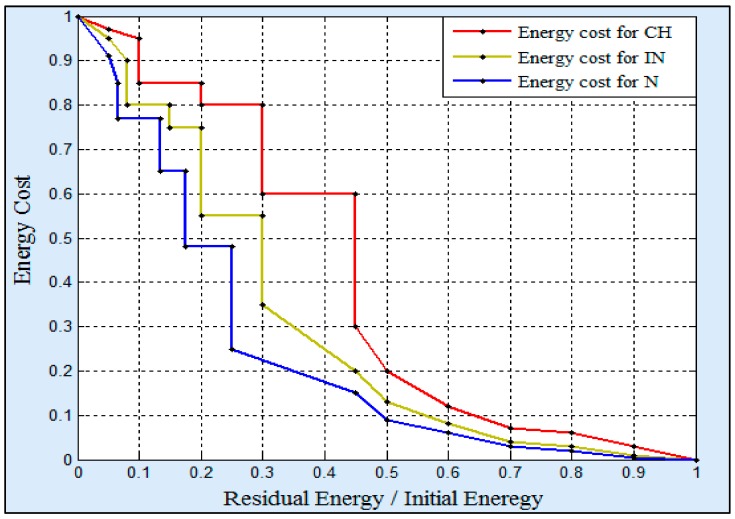
An example of the energy costs for the N, CH, and IN.

The *RE_NHN_* parameter refers to the residual energy in NHN, whereas the *IE_NHN_* indicates the initial energy of NHN. The proposed approach allocates a cost value for every ratio of energy. Figure 7 shows an example of this assumption. Distinctly, for the highest energy ratios have been given the lowest cost values, especially the ratio values that exceed 50% of the initial energy. After calculating the cost values for all the available routes, the N arranges them in its RTs. Thus, the route, which has the lowest cost value is chosen to be used at the beginning of the round. During the round, the Ns of the first level broadcast periodically updated messages in order to update RTs of Ns, which exist in the second level. Based on the updated messages, the Ns update their RTs. Hence, the routes cost values will be dynamically modified. As a result, the route, which was initially selected, may change.

- Inter-clusters Routing

The dynamic approach is also used for routing out of the clusters. Accordingly, all the CHs and INs try organizing their *RT*s. For this reason, they compute the costs for all available routes, which are announced among each other. The cost function comprises of two parts: the first part refers to the cost of the relative position of the next hop (NHI), which might be a CH or IN, to the BS. The second part of the cost function is allocated for the energy cost for the NHI. Thus, the cost value can be calculated using Equation (11) [12]:

(11) $$C_{NHI}\left(n\right)=C_{NHI-BS}\left(n\right)+\alpha \;\cdot \;C_{NHIE}\left(n\right)$$

The *C_NHI-BS_* parameter refers to the distance cost for the NHI. The *C_NHIE_* parameter indicates the energy cost for the NHI. The variable *n* indicates the members of the group of INs and CHs, which have been selected by the source of the data (SNI), either CH or IN, as NHIs in the current round. The first part of the cost function can be calculated using Equation (12) [12]:

(12) $$C_{NHI-BS}\left(n\right)=\frac{{D_{n}}^{2}}{SNI_{D-BS}-NHI_{nD-BS}}$$

Here, the *D_n_* indicates the distance from the SNI to the NHI, whereas the *SNI_D-BS_* and *NHI_D-BS_* parameters denote the distance from the SNI and NHI to the BS, respectively. 

In the second part, an energy cost is given for every remaining energy ratio in NHI. Thus, every energy ratio is replaced with a cost value. Figure 7 shows an example of the energy cost values for the CHs and INs. The higher energy ratios are given lower energy costs, particularly when the energy ratio is overridden 50%. For reducing the energy load on the CHs, it is noticed that the energy ratios of the INs are granted lower cost values in comparison with the CHs. Additionally, the Ns have been given the lowest energy cost values due to their data transmissions are confined within the cluster. After computing the routes costs, the SNI arranges its RT and chooses to send its data through the lowest cost route. During the round, the route costs of the RTs will dynamically change depending on the updated messages which are received from the NHIs. It is worth mentioning that the updated messages are sent periodically. Furthermore, the *SNI* continue sending its data through the lowest cost route.

Thus, the DMR technique provides an approach, which is interested in transmitting the data through the shortest routes, either inside or outside the clusters according to the distances and the energy status of the nodes. Hence, the DMR technique introduces a cost equation for each available route in the source node. The source node sends its data through the route that has the lowest cost. For minimizing the energy load on the next hops, the DMR supposes that the RTs of the source nodes will be updated after receiving updated messages from their members. Based on the cost equation, the source node sends its data through the route that has the lowest cost after updating. By doing so, the DMR technique improves the equilibrium in the network and raises its stability. In addition, it reduces the amount of the energy consumption that is required for the data dissemination.

## 4. Simulation and Results

**Figure 8:**
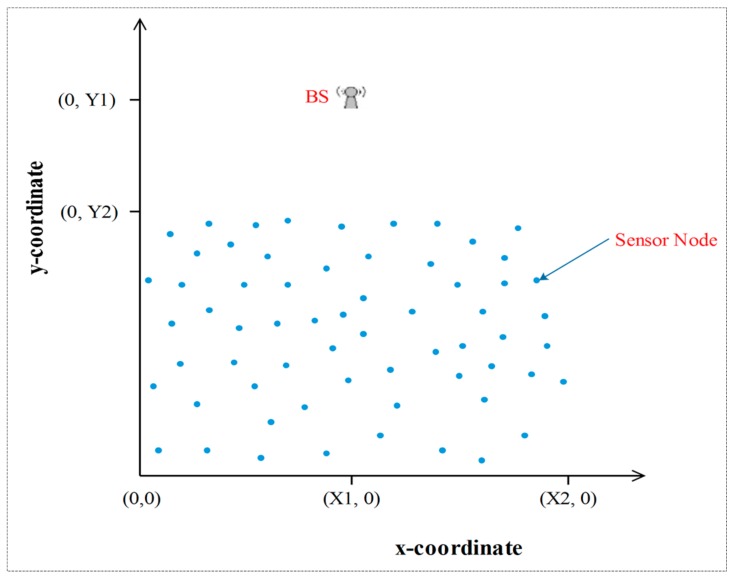
The experimental environment.

In this section, we evaluate the performance of the two techniques that we have proposed in this paper. The experimental results are compared with the ones obtained for the conventional LEACH protocol. Moreover, we extended the comparison to the very much related protocols: the IMHT-LEACH and the EDMHT-LEACH. A MATLAB program is used to evaluate the performance and to make the comparison. The common parameters, which are used in all experiments, are mentioned in Table 1. Initially, we suppose that a fixed number of stationary sensor nodes are randomly scattered on three deployment areas that are different in terms of sizes and distances to the BS; The BS is located out of the sensors deployment area (Figure 8). Table 2 includes the essential parameters of the first experiment.

**Figure 9:**
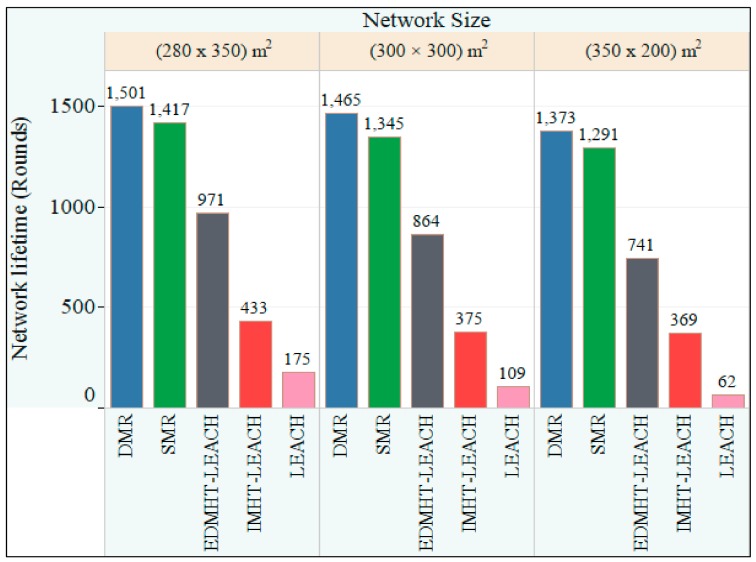
The comparison of the network lifetime versus the network size in the Dynamic Multi-Hop Routing (DMR), Static Multi-Hop Routing (SMR), Enhancing DMHT-LEACH (EDMHT-LEACH), Improved MHT-LEACH (IMHT-LEACH) and conventional LEACH approaches.

Figure 9 represents the network lifetime for the three cases mentioned in Table 2. Based on the obtained results, it can be noted that the two proposed techniques, SMR and DMR, achieved the longest network lifetime in all cases compared with the other protocols. The previous result has been achieved because the proposed approaches suppose that all the scattered nodes on the deployment area are distributed on more levels around the BS and the CHs. Particularly, the CHs and the INs determine their levels around the BS, whereas the Ns of each cluster will identify their levels based on the distances to its CH. Thus, all sensor nodes of the network use multi-hop routing to deliver the data to the destinations either the BS or the CHs. 

In addition, we have noted that the DMR has achieved a longer network lifetime compared with the SMR protocol in all cases. The reason that may be the base of this result is that the DMR technique allows to all the sensor nodes of the network to use different routes within any round. This way it reduces the energy load on the next hops if we compare it to the one that is produced if they stay using the same route, as in case of the SMR technique. Finally, it is observed that the size of the deployment area and its distance to the BS play an important role in the network lifetime. Obviously, the increase in the size of the deployment area and its distance to the BS will decrease the lifespan of the network. It is clear that in such situation, the data transmission distances inside the deployment area and from it to the BS will be increased which causes an increase in the total energy consumption of the network.

In the second experiment, we use a fixed size of the deployment area, while changing the number of sensor nodes that are deployed to this area. Table 3 includes the essential parameters of this experiment.

**Figure 10:**
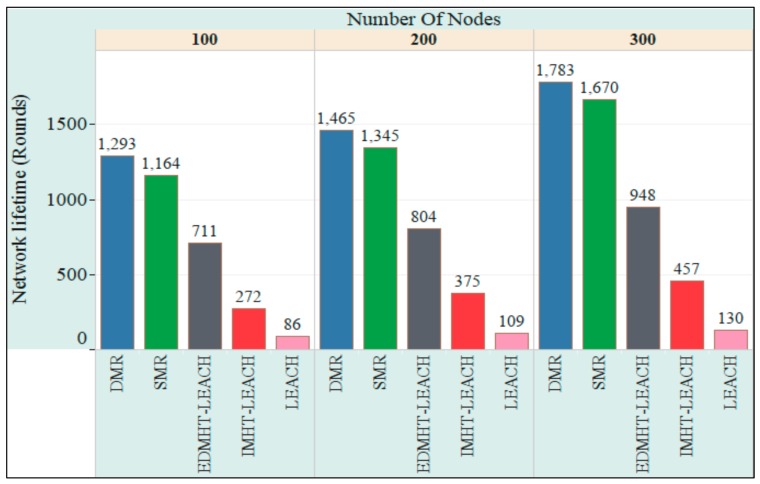
The comparison of the network lifetime versus the number of nodes in the DMR, SMR, EDMHT-LEACH, IMHT-LEACH and conventional LEACH approaches.

Figure 10 shows the network lifetime for the three cases considered in the second experiment. A conclusive comparison can be made, from the lifetime point of view, between DMR, SMR, LEACH, IMHT LEACH and EDMHT LEACH protocols. It can be remarked that the increase in the number of sensor nodes will rise up the lifetime of the network. At the same time, the proposed approaches have prolonged the network lifetime compared with other protocols. This result has been obtained due to the fact that the leveling approach for the whole network, which is used by the DMR and SMR techniques, has contributed in minimizing the energy dissipation in the sensor nodes. This effect becomes more pregnant if it is associated with an increase in the number of nodes that increases the total energy available in WSN. Additionally, it can be noted that the DMR technique has achieved better results than the SMR technique. It is clear that considering the energy status of the next hops, which is proposed by DMR, before transmitting the data has enabled the DMR technique to avoid the rapid decline in the energy of the network’s nodes and maintained the overall balance in the sensor network more than SMR. Hence, the DMR significantly extends the lifetime of the network, particularly if the number of sensor nodes in the network is large. 

To study the effect of the proposed approaches on the network from other points of view, we selected to analyze the experimental results obtained in case of a network which has 200 sensor nodes deployed randomly over an area of (300 m × 300 m). The BS has the coordinates (150 m, 450 m).

**Figure 11:**
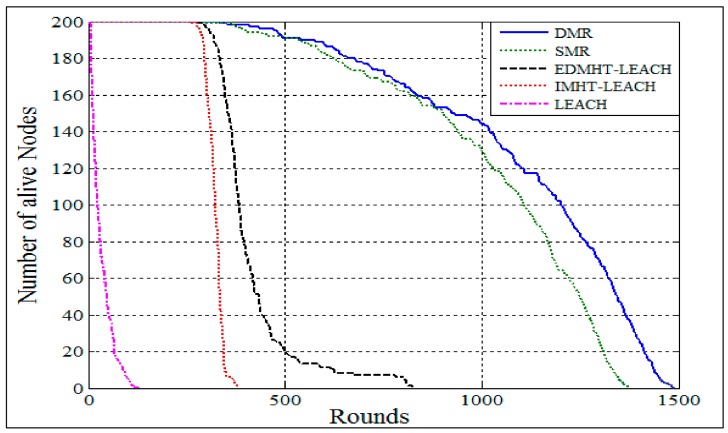
The comparison of the number of alive nodes in the DMR, SMR, EDMHT-LEACH, IMHT-LEACH and conventional LEACH approaches.

Figure 11 shows the relationship between the number of living nodes versus the rounds. It can be seen that the two techniques, SMR and DMR, have achieved a moderated decline in the number of nodes that die versus the rounds in comparison with a very rapid decline presented by other protocols. Hence, the results revealed that the energy dissipation has been gradually reduced by the proposed approaches with a preference for the DMR technique. It should be noted that the main reasons for this result are related to the features of the proposed approaches that have been mentioned previously in this section.

**Figure 12:**
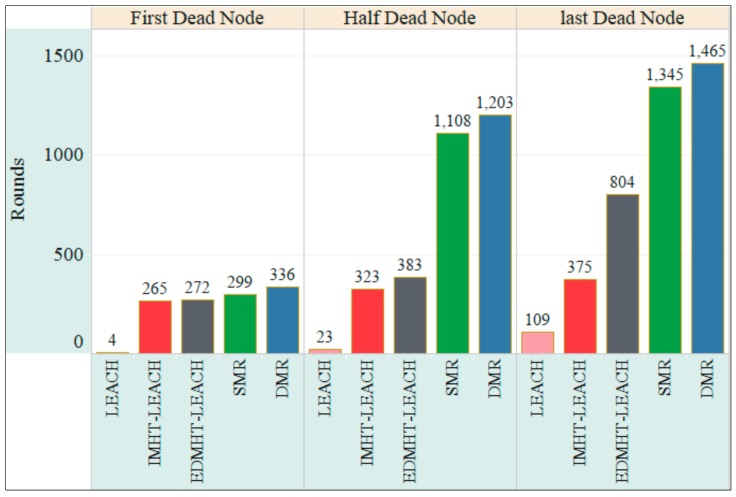
The stability of the network in the DMR, SMR, EDMHT-LEACH, IMHT-LEACH and conventional LEACH approaches versus the rounds.

In Figure 12, the stability of the network, which refers to the first, half and last dead node versus the number of rounds, has been represented for the same set of protocols. Once again, the proposed techniques have achieved the best results compared with the others. Furthermore, the DMR technique has achieved the best result compared with the SMR approach. Here, the moderate receding in the energy of nodes, which is achieved by using the proposed approaches, has contributed in improving the stability of the sensor network. Consequently, the performance of the WSN will significantly increase by using the proposed approaches. On the other side, the dynamic approach, which is based on changing the routes of the data to the BS and the CHs within the same round, has appreciably reduced the energy receding in the sensors network compared with SMR protocol. Accordingly, the DMR protocol enhanced the stability of the sensor network.

**Figure 13:**
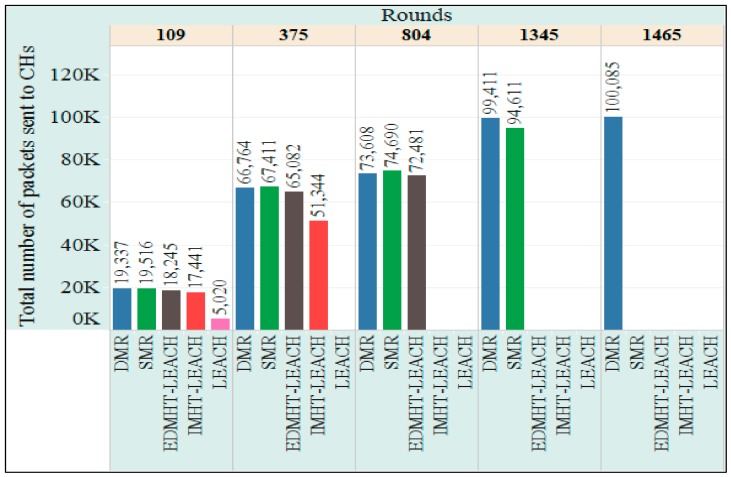
The total number of packets sent to CHs in the DMR, SMR, EDMHT-LEACH, IMHT-LEACH and conventional LEACH approaches versus the rounds.

The throughput of the network is also evaluated. We used two metrics in order to measure the network throughput: the total number of packets sent to the CHs and the total number of packets sent to the BS. Figure 13 shows the total number of packets, which has been sent to the CHs versus the rounds. Clearly, the proposed techniques have obtained the highest number of packets in comparison with the other protocols, with a preference to the DMR technique on the SMR technique. The results mentioned previously have been achieved because the proposed protocols managed to extend the network lifetime and to improve the stability of the network. Accordingly, the number of rounds has increased, which in turn raises the chances of forming more clusters during the network lifetime. As a result, the total number of data packets sent to the CHs will increase. In this context, it is also observed that the DMR technique has obtained better results than the SMR technique. This result has been obtained due to the fact that DMR achieved better results related to the lifetime and stability of the network.

**Figure 14:**
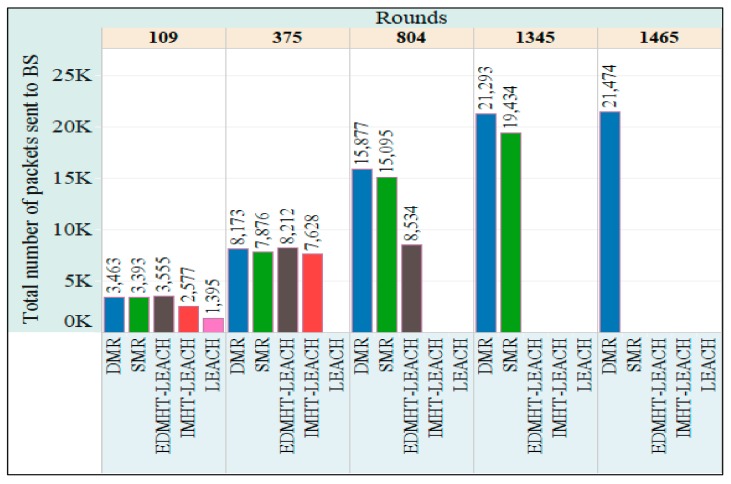
The total number of packets sent to Base Station (BS) in the DMR, SMR, EDMHT-LEACH, IMHT-LEACH and conventional LEACH approaches versus the rounds.

In Figure 14, the total number of packets sent to the BS per rounds is represented. Once more, the SMR and DMR techniques have achieved the best results. The DMR technique outperformed the SMR technique. Once again, improving the lifetime and the stability of the network, which were achieved by using the proposed approaches, plays a key role in increasing the total number of packets sent to the BS. Consequently, the total number of packets, which were sent to the BS from the CHs and the INs, has risen up. 

## 5. Conclusions

In this paper, we have introduced and analyzed two new techniques for routing the data over WSNs: the DMR and the SMR techniques. We assumed that all nodes could be in one of three situations during the network rounds: CH, IN or N. Moreover, this paper has proposed a new topology for the WSN that depends on the area leveling. Hence, all nodes of the network have been distributed into levels. Next, we remarked that there are two types of the data routing: the first type is the intra-cluster data routing and the second type is the inter-cluster data routing. The DMR and SMR techniques have presented different approaches for disseminating the data through the network levels. To evaluate the proposed techniques, a number of experiments have been conducted. The results of the experiments were collected, analyzed and compared with the ones obtained for other related protocols: the EDMHT-LEACH, the IMHT-LEACH and the conventional LEACH.

The results demonstrated that the two new techniques have prolonged the lifetime of the network in comparison with other protocols. The reduction in the data transmission distances, which is obtained by applying the proposed approaches, has minimized the energy dissipation in the network and thus extended the network lifetime. Moreover, the results showed that the proposed approaches have also improved the stability of the network. In fact, the slower decline in the energy of the sensor nodes, which has appeared using the proposed approaches during the lifespan of the network, has increased the stability of the network. The throughput of the network is also evaluated and compared. Once again, the results revealed that the proposed approaches have achieved the best results in comparison with other protocols. The distinctive results, which have been achieved for the lifetime and stability of the network, have raised the throughput of the network. 

Finally, it should be noted that the simulations results have shown that the DMR technique improved the performance of the network more than the SMR technique. The explanation of the previous result is that the SMR technique uses the same data routes during the round, whereas the DMR technique provides a dynamic approach to switching between routes. Accordingly, the energy consumption is reduced by the DMR technique. Thus, the DMR technique obtained better results than the SMR technique in all the evaluation metrics.

In the future, we hope to make several improvements on the current work. For example, we intend to develop a new approach to reduce the duplicate packets, which are produced by the neighboring nodes and transmitted between the levels to the CHs or to the BS. As a result, this may reduce the total energy consumption in the network. At the same time, we shall study a new approach that can give priority levels for each packet in order to be transmit rapidly over the network levels to the BS packets coming from real-time applications.

## Figures and Tables

**Table 1:** The common simulation parameters.

Parameters	Values
BS coordinates (*X*1, *Y*1)	(150 m, 450 m)
Initial energy (*Eo*)	0.5
The percentage of *CH* (*p*)	0.2
Relative weight (*α*)	100
*E_elec_*	50 nJ/bit
*ε_fs_*	10 pJ/bit/m^2^
*ε_mp_*	0.0013 pJ/bit/m^4^
*T_min_*	0.03
Data aggregated energy (*E_DA_*)	5 nJ/bit
Control packet size	200 bit
Data packet size	6400 bit

**Table 2:** The essential simulation parameters of the first experiment.

Number of Nodes	BS Coordinates (*X*1, *Y*1)	Environment Dimensions (*X*2 × *Y*2)
200	(150 m, 450 m)	(280 m × 350 m)
200	(150 m, 450 m)	(300 m × 300 m)
200	(150 m, 450 m)	(350 m × 200 m)

**Table 3:** The essential simulation parameters of the second experiment.

Number of Nodes	BS Coordinates (*X*1, *Y*1)	Environment Dimensions (*X*2 × *Y*2)
100	(150 m, 450 m)	(300 m × 300 m)
200	(150 m, 450 m)	(300 m × 300 m)
300	(150 m, 450 m)	(300 m × 300 m)
